# Local Scale Comparisons of Biodiversity as a Test for Global Protected Area Ecological Performance: A Meta-Analysis

**DOI:** 10.1371/journal.pone.0105824

**Published:** 2014-08-27

**Authors:** Bernard W. T. Coetzee, Kevin J. Gaston, Steven L. Chown

**Affiliations:** 1 Centre for Invasion Biology, Department of Botany and Zoology, Stellenbosch University, Stellenbosch, Western Cape, South Africa; 2 School of Biological Sciences, Monash University, Melbourne, Victoria, Australia; 3 Environment and Sustainability Institute, University of Exeter, Penryn, Cornwall, United Kingdom; University of Colorado, United States of America

## Abstract

Terrestrial protected areas (PAs) are cornerstones of global biodiversity conservation. Their efficacy in terms of maintaining biodiversity is, however, much debated. Studies to date have been unable to provide a general answer as to PA conservation efficacy because of their typically restricted geographic and/or taxonomic focus, or qualitative approaches focusing on proxies for biodiversity, such as deforestation. Given the rarity of historical data to enable comparisons of biodiversity before/after PA establishment, many smaller scale studies over the past 30 years have directly compared biodiversity inside PAs to that of surrounding areas, which provides one measure of PA ecological performance. Here we use a meta-analysis of such studies (N = 86) to test if PAs contain higher biodiversity values than surrounding areas, and so assess their contribution to determining PA efficacy. We find that PAs generally have higher abundances of individual species, higher assemblage abundances, and higher species richness values compared with alternative land uses. Local scale studies in combination thus show that PAs retain more biodiversity than alternative land use areas. Nonetheless, much variation is present in the effect sizes, which underscores the context-specificity of PA efficacy.

## Introduction

Nearly 12% of the world’s terrestrial surface is now classified as some form of protected area (PA) [1]. Indeed, the designation and maintenance of PAs are considered key global strategies to address the growing extinction crisis [1]. The unprotected world has been so transformed by human activity that it can now be characterized more readily by a set of human biomes than by the classic biogeographic regions [2]. Therefore, affording an area protection, a long-standing and current centrepiece of conservation strategy [3], appears to be an effective means of conserving its biodiversity features. Conservation scientists have rightly been concerned, however, that simple assumptions of positive ecological performance may be misleading [4]–[9]. Studies have recognized that effective PA management is key to biodiversity protection [7], and demonstrated that PA designation achieves good conservation return on investment at a relatively low cost [10]. Evidence exists, however, that in many cases PA systems are inefficiently planned to maximize benefits to biodiversity often owing to their spatial location [4], [6], and worrying declines in biodiversity even within PAs in particular regions have been identified [5], [8].

Much interest has focused on determining PA effectiveness in terms of preventing landscape cover changes (e.g. [11]–[13]), but these assessments serve only as a proxy for PA performance, as the measures used cannot necessarily capture the implications of land use change for biodiversity features. Where the latter are investigated, outcomes are typically available for specific areas, such as the tropics [8], or particular taxa, such as birds [14] or mammals [5]. Given that negative pressures on biodiversity and evidence for population declines are global in extent [15], [16], the overall significance of terrestrial PAs for maintaining biodiversity values thus remains unclear. Protected area policy demonstrating their efficacy should ideally be evidence-based, that is, informed by rigorously established objective scientific evidence, as should be the case for conservation policy generally [17]. However, such evidence is not as well developed as it should be [18], despite urgent calls for so doing both in the scientific [18], [19] and policy [3] arenas.

Protected area ecological performance would best be assessed by determining for every established PA what the overall biodiversity status is compared with what would have happened in the absence of protection. Plainly such a comparison cannot readily be achieved. One experimentally tractable alternative is the assessment of biodiversity before and after land cover change, but such studies are extremely rare (although see [20] for a notable exception). The scarcity of data to enable comparisons before/after PA establishment almost invariably necessitates comparisons of each PA with some other area that is unprotected, but similar in all but this designation. As a consequence, assessments of PA performance are typically restricted to small spatial scales and particular taxa (e.g. [14], [21]), but there is no clear indication of the generality of their often-contrasting outcomes [18], [21], [22]. The biodiversity response when comparing a PA with some other area that is unprotected can vary widely, with different studies finding both higher and/or lower biodiversity values across areas [8], [20]–[22]. Results from such studies suggest that PA ecological performance is context-specific and can be influenced by several local factors [8], [18], [22]. As a consequence, the generality of PA efficacy in maintaining biodiversity across regions remains unclear.

Here we use local scale studies comparing biodiversity between PAs and surrounding alternative land use areas to test if PAs contain higher biodiversity values than surrounding areas, using a meta-analysis. Specifically, our aim is to assess the ecological performance of terrestrial PAs, compared with areas in close proximity that are not protected, thus outside PAs, using three key biodiversity attributes: the abundances of individual species (hereafter ‘species abundances’), assemblage abundances (summed across species) and assemblage species richness. These are key measures of biodiversity [23]. We also use an information theoretic approach with candidate explanatory variables to explore reasons for the variation in effect size. We then consider sources of bias in interpreting results, and highlight the benefits and shortcomings of our approach in determining PA efficacy.

## Methods

### Literature search

Our search and data extraction protocol follows best practice guidelines in conducting meta-analysis (see Appendix S1). We used keyword searches in Web of Science, Scopus, and Google Scholar for relevant papers published from 1975–2011, and their references, and included those reporting pairwise comparisons of biodiversity measurements either inside and outside protected areas (PAs) or between areas within PAs (details follow). The initial search string was: (bird* OR mammal* OR reptile* OR amphibia* OR arthropod* OR insect* OR fish* OR plant* OR vegetation*) AND (“protected area” OR “protected areas” OR “national park” OR “national parks” OR “reserve” OR “reserves” OR “game reserve” OR “game reserves”) AND (effective* OR inside* OR performance* OR assessment* OR evaluation* OR estimate* OR comparison* OR contrast*) AND (outside* OR adjacent* OR neighbour* OR adjoining* OR bordering* OR near*). We also used unstructured and opportunistic literature searches with sections of the initial string (particularly to identify studies on different taxonomic groups), expert knowledge of available data sets, and results of a recent multi-database systematic review on protected area efficacy [9]. Grey literature (informally published written material [such as reports, theses and books]) was targeted but exceedingly rare, as found by others [9].

### Data capture

From suitable papers we retained studies that measured biodiversity responses inside PAs, and outside PAs, in a replicated study design. These data represent three response variables: (i) species abundances (the abundances of individual species), (ii) abundances per assemblage, (iii) species richness per assemblage, following [24]. Species abundance represents indices of abundance; for example counts, density, capture frequency, occupancy estimates and biomass, for a single species both inside and outside PAs, but only in cases where taxonomy was resolved to the species level (species included are listed in the Dataset S1; N = 243). Assemblage abundance represents indices of abundance; for example counts, density, capture frequency, occupancy estimates and biomass, from cases where abundance was reported across species assemblages or could be calculated across sampling sites. We included these data as estimated by the original authors, but ensured that authors followed a replicated study design, and identical calculations of such indices both inside and outside PAs, and so we consider this response variable as an additional indicator of abundance across species groups (following [24]). Species richness included, for example, observed/estimated/rarefied richness, species density and genera/family richness, from cases where richness was reported or could be calculated across sampling sites. The use of these three variables as estimates of biodiversity was constrained by the approaches adopted by the studies we examined. Although biodiversity can be measured in a variety of ways, these three measures are commonly used as effective measures of biodiversity [23].

Most data we used included mean, standard deviation and sample sizes both inside and outside PAs. Where standard deviation was not reported (20%; 300/1484), we calculated it from either standard error or confidence intervals using imputation methods [25]. Studies with no suitable variance measures were omitted. Where the species could not be correctly identified to species level, data were omitted from the species abundance analysis, but included in the assemblage abundance analyses in cases where these values could be summed across groups of unidentified species. Invasive and domesticated species were omitted as they are not considered here to be of conservation interest. Observations on presence or absence of species were omitted as effect size cannot be calculated. Where data were reported across mixed habitats and/or vegetation types, we only took those from matched pairs, i.e. in areas where the same habitat type was reported. To avoid inadequate treatment of fragmentation effects, data from habitat fragments, typically forest fragments, were excluded following [24]. We did not find studies comparing biodiversity features before and after PA establishment. Few studies considered census-area effects [26] directly, but as individual studies had similar (often identical) sampling designs inside and outside the PAs and in consequence comparable sampling areas, we consider the potential confounding effects of sampling area negligible. Data reporting diversity indices were omitted to avoid pseudoreplication [24], as they are secondary (or derived) measures of species richness and/or abundance, and data on demographics or community structure were omitted because the direction of the expected response was not straightforward to interpret [24], [27]. WebPlotDigitizer v2.4 [28] was used to capture data from figures, which is considered a robust technique [29].

**Figure 1:**
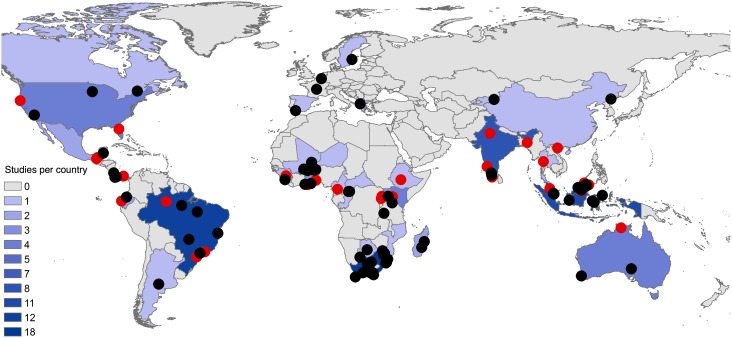
Map of the study sites by the centroid coordinates of protected areas for inside-outside pairwise comparisons (black dots; n = 71) and inside only comparisons (red dots; n = 32). Both categories include data where studies reported across clusters of protected areas.

We found 861 pairwise observations inside and outside PAs from 86 studies distributed amongst 32 countries and 57 PAs that met our criteria (Figure 1; all studies used in the meta-analysis are in Dataset S1; Figure S1 in File S1). During the search, we also discovered comparisons within PAs only, that typically included a pristine baseline site (as judged by the authors) in the PA and an anthropogenically disturbed area also inside the PA (disturbances such as logging, clearing or hunting pressure). To determine if PA designation may offset negative anthropogenic influences, such as land transformation, we compared this portion of the dataset to the comparisons made inside and outside PAs. If the effect sizes of comparisons between areas within PAs are lower than for comparisons made inside and outside PAs, we can infer that PAs offset negative anthropogenic influences, such as land transformation, to a greater degree than no PA designation. We identified an additional 623 such pairwise comparisons from 41 studies between sites within PAs only.

### Data analysis

To estimate effect size, we calculated the Hedges *g* metric for pairwise comparisons. This is the weighted average of the mean standardized difference (based on pooled variance measures). The metric is the most commonly used, and preferred, to compare pairs of means where variance is available, and unlike others is insensitive to unequal sampling variances in paired groups [27], [30]. It is defined as
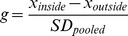
where



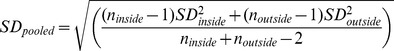



Since Hedges *g* is a biased estimator of population effect size [27], [30], we used the commonly applied conversion factor *J* to compute the bias-corrected Hedge’s g* metric [27], or g* = *gJ*, where
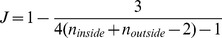



We then calculated effect sizes using a random-effects model that weights individual comparisons by the inverse of within-study variance plus between study variance, following [24], [27], [30], with a maximum likelihood variance estimator. Random effects models encompass the variance both between-studies and within-studies, and as such, they are the most appropriate for the majority of ecological meta-analyses because they account for variation in study-specific effects [27]. All analyses were conducted in the “metafor” package [31] in R [32].

We set the direction of the sign of the effect size as positive when the biodiversity value for PAs was greater than outside PAs, implying that PAs contain higher biodiversity values [33]. We selected a single effect size measure that could incorporate variance, which was standardized across response variables. We calculated effect sizes for pairwise comparisons across data inside and outside PAs (N = 861), and also for comparisons inside PAs only (N = 623). We then calculated effect sizes for the three response variables. For inside and outside comparisons only we further calculated effect sizes for (i) five major taxonomic groups (mammals, birds, herptiles [reptiles and amphibians combined], arthropods, plants), (ii) continents (excluding Antarctica with no data), and (iii) International Union for Conservation of Nature’s Protected Area Management Categories (a globally recognised PA categorisation system [34]). The categories are primarily based on their management objectives, in which categories 1–4 reflect stricter goals for biodiversity conservation [1 being the strictest], and 5–6 generally allow extractive use via exploitation of biodiversity features [34], see full definitions in Appendix S2). Finally, (v) we calculated effect sizes for the status of species on the IUCN Red List (a global inventory of the threat status of species according to predetermined criteria [35]).

We calculated two commonly used metrics to characterize heterogeneity between pair-wise comparisons as employed in the “metafor” package [31] in R [32]: the Q-statistic and the *I* ^2^ value. Total heterogeneity in effect size can be tested with a Q-statistic where a significant value indicates that the estimated effect size is more heterogeneous than expected by chance [27]. The total heterogeneity of the study, *Q_TOTAL_* or *Q_T_* can be calculated as
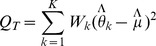



The *I* ^2^ value is presented as the total percentage of heterogeneity that can be attributed to between-study variance [27]. The metric quantifies the heterogeneity by comparing the calculated *Q_T_* to its expected value under the assumption of homogeneity [27], as
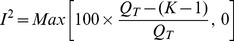



We conducted three commonly used tests for detecting bias in meta-analysis in the “metafor” package [31] in R [32]: funnel plot, cumulative meta-analysis and Orwin’s fail safe N [27], [30], [31]. A funnel plot graphs effect size against standard error, and assumes that studies with the largest sample sizes will have lower standard error, and so will be near the average effect size, while studies with smaller sample sizes will be spread on both sides of the average effect size. Variation from this assumption can indicate bias, although the source of such bias may be unclear from a funnel plot [27]. However, positive asymmetry is typically taken to mean publication bias, in that those studies with positive effects are submitted and/or accepted for publication with a greater frequency then those with negative effects [27]. A cumulative meta-analysis sorts all pair-wise comparisons by precision, thus starting with the studies with the largest standard error, after which the comparison with the next largest standard error is added and the effect size is recalculated, and so continues iteratively [27]. The resulting graph enables inspection of the development of the observed effect size with the addition of more precise data. Orwin’s fails safe N is a metric of the number of studies averaging null results that would have to be added to the observed outcomes to reduce the average effect size to half the observed effect size. All tests for publication bias in this study showed that it was negligible [27] (Table S1; Figure S2 & S3 in File S1).

To address the potential spatial pseudoreplication in the dataset that could arise from multiple responses reported within studies [22], PAs, countries or species, we recalculated effect sizes after sampling one pairwise comparison only per study, PA, country or species, respectively. This resampling was repeated 10 000 times for each of these four parameters and the estimated mean and 95% confidence intervals compared with the overall effect size, which was conducted in R [32].

We used an information theoretic approach [36] to assess the influence of a candidate set of models and variables to explain the variation in effect size, where data were available for all variables. Models tested the influence of (i) pre-planned subgroups in the meta-analysis (variables: response variable, taxonomic group, PA IUCN Category), (ii) design, location and structural attributes of the PAs (variables: continent, latitude, longitude, PA area in km^2^, and PA establishment date, using [34]) and (iii) influence of socio-economic conditions of the countries in which PAs are located (variables: World Governance Index [37], Gross Domestic Product (GDP), Country Population size and the Gini coefficient of income inequality [38]). We followed an exhaustive search approach, which entails fitting all possible model formulations, with a Generalized Linear Model (GLM; [39]). We assumed a Gaussian distribution with a log link function, which was identified as the appropriate family and link function by visual inspection of quantile-quantile plots, using the “glmulti” package [40], in R [32]. We selected the highest ranked model based on the lowest Akaike Information Criterion [39], [40] value. Furthermore, to address possible pseudoreplication, one pairwise comparison per study was selected at random and the respective GLM model refitted as above. We selected the highest ranked model based on the lowest Akaike Information Criterion value [39], [40], and repeated this procedure 1000 times, to calculate the proportionally highest ranked model for each candidate dataset.

At least some of the variation in effect size may also be accounted for by the scale over which studies were conducted. To test the influence of distance between PA boundaries, and the maximum distance between pair-wise comparisons, a best GLM model by exhaustive fit was performed as above [40]. These independent variables were (i) the maximum distance to protected area boundary within studies, and (ii) the maximum distance between pair wise comparisons within studies, meaning, within each study, the maximum distance between sampling points assigned to all points in that study. Since the data on the distance between comparisons were only available for a reduced subset of pair-wise comparisons (N = 569), we performed a separate GLM as above with only the distance variables. We also estimated Pearson’s product moment correlation coefficient between Hedges g* metric values for pair-wise comparisons and the two distance variables in R [32].

## Results

**Figure 2:**
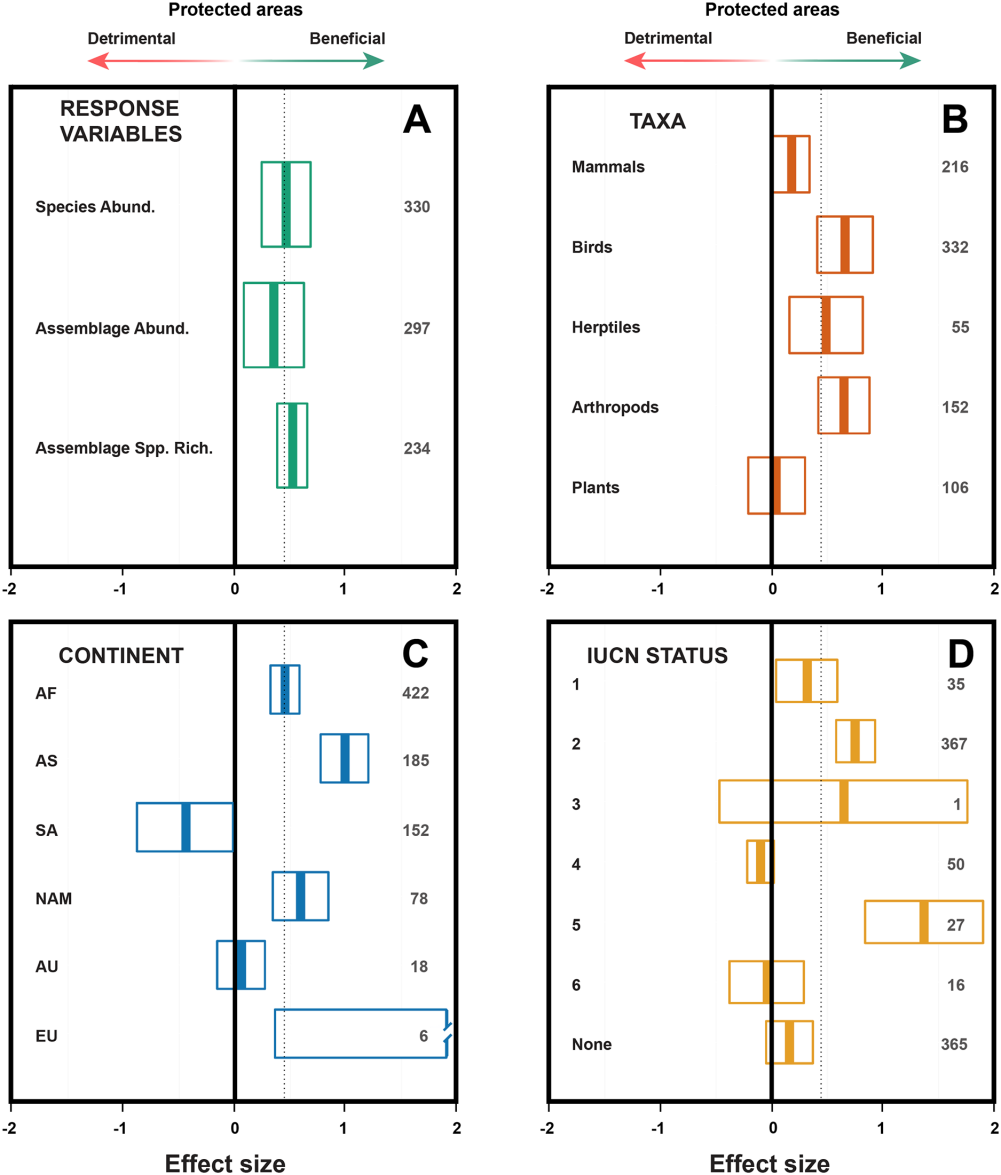
Effect sizes and 95% confidence intervals of response variables (A), taxa (B), continent (C) and the Protected Areas IUCN category (D). Positive boxplot values indicate a net positive impact of protected areas (PAs) on biodiversity. Sample sizes are in grey, the vertical black lines show a zero effect size, while the dashed lines show the overall effect size of 0.444. The effect size for the truncated bar (Europe; Panel C) with large variance due to low sample sizes is 2.54 CI: 0.36–4.73. Abund = Abundance; Spp. Rich.  = Species richness; AF = Africa; AS = Asia; SA = South America; NAM = North America; AU = Australasia; EU = Europe. IUCN categories are detailed in Appendix S2.

The mean effect size using the random effects model, which provides an indication of the general trend across all 861 comparisons, was 0.444 (95% confidence intervals 0.324–0.564; Table 1). Substantial variation was present in the direction and size of effects in response variables for different pairwise comparisons (I^2^>87.9%; Q-statistic significant at <0.01; Table 1). However, when fitting the random effects model, PAs had higher species abundances (Figure 2A; N = 330), assemblage abundances (Figure 2A; N = 297) and assemblage species richness (Figure 2A; N = 234) than land use areas outside PAs (see Table 1).

**Table 1:** Effect sizes (ES), lower bound (lbCI) and upper bound (ubCI) confidence intervals and sample sizes (N), Tau square (I^2^), Q statistic (Q) with its p-value (Qp) for at different subgroup designations.

Description	Subgroup	ES	lbCI	ubCI	N	I^2^	Q	Qp
Overall	Inside Outside PAs	0.444	0.324	0.564	861	94.9	8620.1	<0.01
Overall	Comparisons insidePAs only	0.172	0.083	0.261	623	46.8	1439.9	<0.01
Overall	PAs with IUCNdesignation	0.621	0.488	0.755	496	92.9	3622.3	<0.01
Overall	PAs with no IUCNdesignation	0.161	–0.050	0.372	365	95.9	4910.5	<0.01
Overall	Clusters of PAs	0.560	0.400	0.719	189	86.1	980.9	<0.01
Overall	Unique PAs identified	0.413	0.264	0.562	672	95.8	7638.9	<0.01
Variable	Species abundance	0.517	0.382	0.652	330	87.9	1869.6	<0.01
Variable	Assemblage abundance	0.349	0.083	0.615	297	97.6	4997.7	<0.01
Variable	Assemblage speciesrichness	0.457	0.238	0.676	234	93.2	1666.9	<0.01
Variable	Species abundance(inside)	−0.086	−0.235	0.063	295	50.2	721.4	<0.01
Variable	Assemblage abundance(inside)	0.165	0.044	0.286	152	9.9	270.8	<0.01
Variable	Assemblage speciesrichness (inside)	0.529	0.364	0.694	176	51.7	418.5	<0.01
Taxon	Mammals	0.179	0.010	0.344	216	88.2	1129.6	<0.01
Taxon	Birds	0.657	0.410	0.910	332	97.2	5415.6	<0.01
Taxon	Herptiles	0.487	0.159	0.816	55	89.6	425.4	<0.01
Taxon	Arthropods	0.654	0.421	0.882	152	64.1	406.1	<0.01
Taxon	Plants	0.043	−0.214	0.301	106	95.4	1167.8	<0.01
Continent	Africa	0.450	0.319	0.581	422	92.9	3320.0	<0.01
Continent	Asia	0.986	0.769	1.203	185	71.2	571.4	<0.01
Continent	South America	−0.441	−0.883	0.002	152	98.2	3950.6	<0.01
Continent	North America	0.587	0.340	0.835	78	84.0	387.2	<0.01
Continent	Australasia	0.056	−0.160	0.273	18	4.2	16.9	<0.01
Continent	Europe	2.544	0.362	4.726	6	97.1	133.3	<0.01
IUCN Cat.	IUCN 1	0.316	0.038	0.594	35	0.0	32.1	0.560
IUCN Cat.	IUCN 2	0.754	0.581	0.926	367	94.8	3204.3	<0.01
IUCN Cat.	IUCN 3	0.646	−0.473	1.764	1	NA	0.0	0.999
IUCN Cat.	IUCN 4	−0.099	−0.222	0.025	50	42.1	104.5	<0.01
IUCN Cat.	IUCN 5	1.371	0.838	1.904	27	83.8	112.6	<0.01
IUCN Cat.	IUCN 6	−0.044	−0.382	0.295	16	0.0	6.1	0.978
IUCN Cat.	No IUCNDesignation	0.161	−0.050	0.372	365	95.9	4910.5	<0.01
Red List	Not Evaluated	0.596	0.234	0.957	62	91.0	402.8	<0.01
Red List	Data deficient	−0.479	−1.170	0.212	6	85.2	32.4	<0.01
Red List	Least Concern	0.460	0.291	0.628	168	83.4	744.8	<0.01
Red List	Near Threatened	1.027	0.262	1.791	27	95.2	323.8	<0.01
Red List	Vulnerable	0.814	0.333	1.294	28	89.2	195.4	<0.01
Red List	Endangered	0.557	0.266	0.848	22	60.9	50.1	<0.01
Red List	CriticallyEndangered	−0.119	−0.548	0.311	6	66.3	14.5	0.013
	Small mammals	0.042	−0.236	0.320	25	3.4	21.0	0.637
	Large mammals	0.372	0.131	0.613	114	93.1	796.6	<0.01

PA = Protected area. Cat = Category.

Effect sizes for PAs with no IUCN category designation were lower than those with a designation, but remained positive and overlapped with the overall effect size and so we included them here (Table 1). Studies that reported across clusters of PAs rather than individual PAs remained positive and were thus included in the overall assessment of effect size (Table 1). When resampling effect sizes to account for pseudoreplication, they remained positive and overlapped with the overall effect size for both inside-outside PAs and inside PA only comparisons, but were less positive for species responses (Table S2). The variance of these resampled effect sizes also increased, but we note that effect size precision increases with additional data (Figure S3 in File S1). Thus, the present results overall can be considered robust to pseudoreplication.

Although variable, the mean effect sizes confirm that on average PAs contain significantly higher numbers of species and more individuals for mammals, birds, herptiles and arthropods, but the effect is non-significant for plants (Figure 2B; Table 1). Small mammals showed a smaller effect size for species abundance (<1 kg; N = 25; 0.042; CI: −0.236–0.320) than did large mammals (>1 kg; N = 114; 0.372; CI: 0.131–0.613). These results suggest that while most species benefit from PA establishment, a suite of them, typically plants, fare better outside PAs in typically anthropogenically transformed habitat.

Protected area efficacy by continent generally showed positive effect sizes, apart from South America (strongly negative and only just non-significant) and Australia (also non-significant; Figure 2C). We note that sample sizes for Europe and Australia are low.

Improved biodiversity outcomes with an increase in IUCN management category would seem an obvious *a priori* outcome, but such a simple relationship was not clear (Figure 2D). IUCN Category 2 PAs, and to a lesser extent Category 1 PAs, had a high positive effect size. However, although the sample sizes are low, so did IUCN Category 5 PAs that allow much extractive use within their borders. PAs with no IUCN designation, and those of categories 4 and 6 had no significant effect and few data are available on Category 3 PAs.

**Figure 3:**
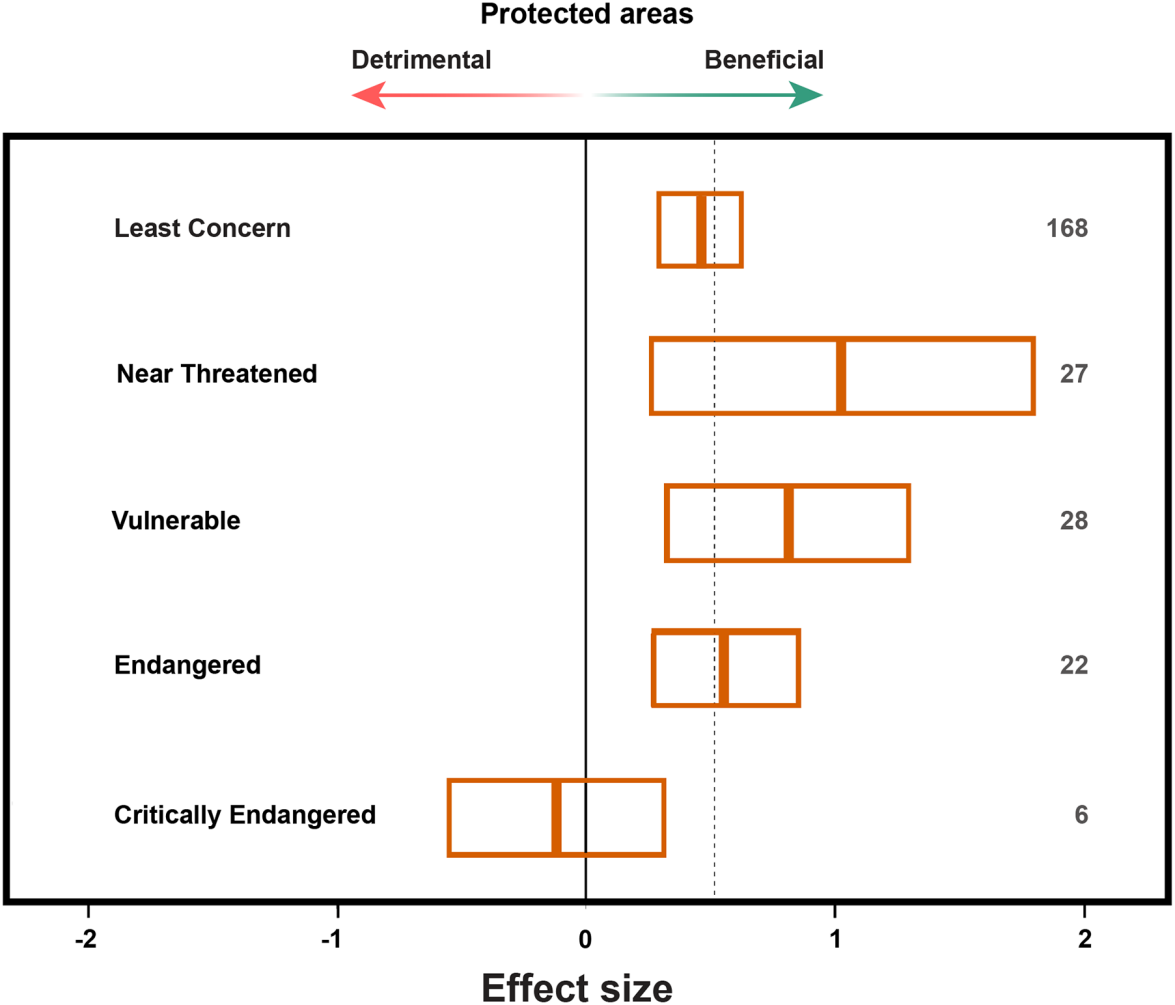
Effect sizes and 95% confidence intervals for species abundance responses by their IUCN Red List status. Positive boxplot values indicate a net positive impact of protected areas on species abundance. Note that multiple responses may be reported for one species (unique species = 168, total cases = 251) and taxonomic uncertainties, Not Evaluated and Data Deficient species are excluded (n = 79). The vertical black line shows a zero effect size while the dashed line indicates the overall effect size for species abundance responses (0.517). Taxa included mammals, birds, herptiles and plants.

Species listed as Least Concern, Near Threatened, Vulnerable or Endangered by the IUCN generally had greater abundances inside than outside PAs (Figure 3; Table 1). A small sample size of only six observations from one species (*Gorilla gorilla*) that is Critically Endangered was non-significant. However, at least for species abundance, few data on those species of greatest conservation concern, as measured by their Red List status, are available (Figure 3). Indeed, published studies comparing the abundances of highly threatened species both inside and outside PAs are rare, and many such species do not occur in PAs [18], [41].

The overall effect size from pairwise comparisons inside PAs only (0.172; 95% confidence intervals: 0.083–0.261) was lower than that of the inside and outside comparisons only (their 95% confidence intervals did not overlap). When fitting the random effects model, pristine areas in PAs only, did not have significantly higher species abundances (−0.086; CI: −0.235–0.063; n = 295), but did have significantly higher assemblage abundances (0.165; CI: 0.044–0.286; n = 152) and significantly higher assemblage species richness (0.529; CI: 0.364–0.694; n = 176) than anthropogenically disturbed areas.

The variation explained by the fitted explanatory models was low, with the meta-analytical and socio-economic models each accounting for about 5% and 7%, respectively, of the variation in effect size (Table S3). By contrast, the PA-model accounted for 25% of the variation (Table S3). For all candidate explanatory variables there were multiple competing best-fit models (Table S4). Distance among comparison sites explained only *c*. 1% of the variation in effect size for studies included in our meta-analysis (Table S5). Indeed, despite being significant the relationship between effect size and the greatest distance between comparison sites was weak (Pearson’s r = 0.146; p<0.001) as was the relationship with distance to PA boundary (r = 0.085; p<0.05).

## Discussion

An initial assessment of global PA efficacy should be to determine if differences exist in biodiversity between PAs and unprotected land in a direction demonstrating higher biodiversity values in the former. Most of the studies included here did indeed find higher species abundances, assemblage abundance, and species richness inside PAs compared to areas outside them. This pattern holds across taxonomic groups (although non-significant for plants) and continents (although non-significant for South America and Australia).

What mechanisms underlie the non-significant effects for plants and for South America and Australia are not entirely obvious. Clearly, habitat change has major effects on plants, with many studies documenting replacement of particular species and changes in habitat structure [2], [24], [42], [43]. Likewise there has been a growing focus on the scarcity of large old trees [44]. The plant studies analysed here included 53 pairwise comparisons of species richness, 41 of assemblage abundance and 12 of species abundances. All of the 12 species abundance studies were concerned with trees. This balance of investigations to date may account for the lack of an effect, and may rather reflect the globally emerging fingerprint for biodiversity loss under global change, where community turnover, but not necessarily species richness change is observed [43]. In the case of Australia, the small sample size may be driving the absence of an effect size. Alternatively, most data from Australia (84%; 16/19) came from only one study that documented the recovery of small mammal populations after their isolation from invasive predators [45], and so it is not clear to what extent this outcome reflects general patterns on the continent. For South America the situation is also difficult to explain. The taxon with the most data for South America was the mammals (53%; 81/152), many of them large (>1 kg; 54%; N = 44), and their increase outside PAs contributes to the non-significant outcome. One explanation may be hunting pressure. Carrilo et al. [46] found that increasing hunting pressure inside a PA diminished large mammal populations in Costa Rica. In contrast to our general finding, Negroes et al. [47] found that private forest reserves in Brazil were responsible for conserving medium to large-sized vertebrates, more so than PAs. While primary forest is globally irreplaceable for conserving biodiversity [24], land use areas under low extraction, or regenerating forests, seem to contribute to a degree to an integrated landscape level conservation strategy, which may be particularly true in South America [48]. Furthermore, in a comparable meta-analysis, Gibson et al. [24] found that primary forests in South America retain more biodiversity than transformed forests, but since they did not focus on PAs exclusively, they had a larger sample size (N = 909). This finding emphasises that greater sample sizes could increase a positive effect size signal between transformed and more natural areas, a finding that our data corroborate (Figure S3 in File S1).

Despite effect sizes generally being positive, PA efficacy clearly varies considerably amongst PAs, species and local contexts, as demonstrated here and in region-specific studies [5], [8], [17], [21]. Determining the mechanisms driving the pattern of higher species abundances, assemblage abundance, and species richness inside compared to outside PAs remains challenging. Using GLMs we sought to explore a range of proximate factors that might explain this variation. We included explanatory variables such as spatial structure, socio-economic conditions, and structural attributes of PAs, which have been shown elsewhere to have effects on biodiversity values [4], [6], [49]–[55]. These assessments provided some insight, but did not account for much of the variation in effect sizes found here. Since comparisons inside and outside PAs in our database were mainly made at the local scale, the geographic context explained little of the observed effect size variation. In consequence, the extent of site matching, which may play a role in increasing estimates of PA efficacy outcomes in some cases [56], may be less important in our database comparing biodiversity features themselves, rather than proxies such as deforestation (but see [56]). Socio-economic factors influence conservation outcomes in some regions [50]–[52], but here also fared relatively poorly at explaining variation. Likewise structural variables such as PA size and location explained little of the variation. Some of these variables are important drivers of biodiversity variation generally, such as area and latitudinal position (mostly via energy availability) [57], [58], [59]. However, they have much less of an influence *per se* on differences in diversity inside and outside PAs. Such an outcome suggests that PA efficacy itself is invariate, at least with regard to these variables. In other words the largest effect is of the PAs themselves, as we found here. Our meta-analysis of studies which compare pristine and transformed areas within PAs themselves bears out this suggestion. The pristine areas typically have higher biodiversity values than the transformed areas. Moreover, the effect size here is weaker than the effect size found when comparisons are between sites inside and outside PAs. Together with evidence that pristine forests retain biodiversity features to a greater degree than transformed areas [24], these outcomes suggest that PAs must offset negative anthropogenic influences to a greater degree than no PA designation.

Nonetheless, additional variation may be attributable to other factors such as characteristics of the organisms themselves. For mammals at least, we were able to show that larger species in particular are reduced outside PAs, in keeping with other evidence that smaller mammals are typically better able to tolerate conditions outside PAs [8], [21], [53], [60]. Others have also highlighted the contrasting responses in biodiversity documented by assessments among PAs, but comparing different taxonomic groups [8], [21]. Interspecific variability in population responses to landscape change is well-known (e.g. [61]), and some taxa obviously fare better in transformed landscapes, even within PAs. Whether this is the case for species that have a high threat status is more difficult to discern given that information for such species is so scarce [41]. Unfortunately, the scope of our data did not enable us to pursue in more detail possible factors underlying effect size variation among continents and other taxa, largely because of the risk of misleading subgroup effects [25], [27]. A range of factors could explain the variation we found given substantial life history differences among these taxa (e.g. dispersal distances, life span, migration propensity, tolerance of disturbance [62]–[64]), and considerable differences in the evolutionary history of species on continents [59], [65].

Similarly, the relationship between the designated management status of a PA and effective conservation of its biodiversity features also seems to be unclear. This outcome provides further evidence for calls that the IUCN PA management categories should be reassessed to reflect biodiversity outcomes rather than management objectives (see [66]). Thus, challenges for conservation science include determining which mechanisms drive positive PA efficacy, under which conditions PAs fail species with differential responses to threats, and concomitantly, IUCN threat designations, and how management categories and biodiversity outcomes can best be aligned.

In addition to these proximate mechanisms that might influence effect size, several ultimate mechanisms may also be important. These include (i) the observation that PAs are non-randomly placed, typically biased towards areas of inaccessibility, which in itself would reduce threatening anthropogenic processes [54]; (ii) the persistence of existing differences in abundance and richness between the areas at the time of PA designations, as a result of the choice of location [18]; (iii) that lower levels of threatening processes, such as habitat alteration or exploitation, have prevailed inside PAs than elsewhere; (iv) that active management of PAs has maintained or increased abundance and richness relative to outside PAs [18]; and/or (v) leakage effects, where threatening processes are displaced from PAs to surrounding areas [55].

Given that data on biodiversity condition before and after establishment of PAs is so rare, studies have understandably had to focus on some comparison of PAs to other areas that may embody likely outcomes if the area had not received PA designation. However, the effect size as calculated here can inherently not explicitly consider the counterfactual (the biodiversity outcome that would have occurred if there had been no PA designation, see [18], [56], [67]). Site matching approaches attempt to address this bias arising when observable biophysical and socioeconomic factors may affect biodiversity in addition to PA designation [56], [67]. While these studies may detect a weaker signal for the influence of PA designation itself, they still typically focus on proxies of biodiversity to determine performance outcomes, such as deforestation offsets. Although the irreplaceability of primary forests for biodiversity has been established [24], measures of deforestation cannot characterize changes in species richness and abundance itself. As a consequence, the vast majority of published studies on biodiversity have taken a broader view of the counterfactual, and made what for the investigators seemed *a priori* sensible comparisons between PAs and other areas that they held to embody likely outcomes if the former had not received designation. However, due to the way the original studies were designed, the dataset developed here cannot be analysed with site matching approaches. Thus some concern might remain that the observed effects have been influenced by any one or many of the mechanisms above. Nonetheless, the ultimate mechanisms that drive patterns of higher biodiversity retention within PAs are clearly far from universal, and their geographic distribution and intensity is poorly known [18], [67]. Therefore, a comprehensive assessment of PA effectiveness can benefit from assessments that consider the net outcomes of both observed effects (as is the case here) in addition to those approaches that can better quantify assessments of effects of bias (i.e. site matching approaches [56] or experimental designs [20]).

In conclusion, despite the ultimate mechanisms underlying our findings not being firmly established, and much variation in effect sizes across regions and among taxa remaining unexplained, a signal is clear: PAs have positive biodiversity values compared with alternative land uses. In consequence, our results, together with emerging qualitative evidence [9], suggest that in general PA establishment itself may confer a net benefit to biodiversity. Thus, our approach provides a quantitative demonstration of the value of PAs as an effective strategy for conserving biodiversity. In other words, studies undertaken at a local scale to date clearly indicate that an ecological foundation exists from which the economic, political and social benefits of PAs are being realized [68]. This outcome provides evidence in support of the value of the Aichi Biodiversity Target 11 of the Convention on Biological Diversity’s (CBD) new Strategic Plan for Biodiversity [69], the nationally agreed goals to fulfil signatory countries commitments under the CBD [69]. Target 11 calls for the expansion and effective management of PAs. The outcomes of our analyses show why this Target is worth achieving.

## Supporting Information

File S1 Contains the following files: **Table S1.** Orwin’s fail safe N is 1238 to reach an overall effect size of 0.222. **Table S2.** Effect sizes determined by resampling one pairwise comparison per unit of study, per species, per country and per protected area (PA), to assess the potential spatial pseudoreplication in our dataset arising from multiple responses. **Table S3.** Best GLM models by exhaustive fit for the Meta Analysis model, Protected Areas (PA) model and Socio-Economic model. **Table S4.** Proportion of five highest ranked models for Meta Analysis model, PA model and the Socio-Economic model. **Table S5.** Best GLM model by exhaustive fit for two variables, the maximum distance to protected area boundary within studies, and the maximum distance between pair wise comparisons within studies. **Figure S1.** PRISMA flow diagram, depicting the flow of information through different phases of the search process conducted. **Figure S2.** Funnel plot of effect size standard error plotted against effect size for all inside-outside pairwise comparisons. **Figure S3.** Cumulative meta-analysis of the dataset sorted by precision, with effect sizes and 95% confidence intervals (n = 861). **Appendix S1.** PRISMA (Preferred Reporting Items for Systematic Reviews and Meta-Analyses) checklist. **Appendix S2.** Detailed descriptions of IUCN protected area management categories.
(DOCX)

Dataset S1 Complete dataset and references included in the meta-analysis.
(XLSX)
