# Supplementary material for: Local Scale Comparisons of Biodiversity as a Test for Global Protected Area Ecological Performance: A Meta-Analysis
Source: PLoS One. 2014 Aug 27;9(8):e105824. doi: 10.1371/journal.pone.0105824 (PMC4146549; doi:10.1371/journal.pone.0105824)
Supplement: File S1 — Contains the following files: Table S1. Orwin’s fail safe N is 1238 to reach an overall effect size of 0.222. Table S2. Effect sizes determined by resampling one pairwise comparison per unit of study, per species, per country and per protected area (PA), to assess the potential spatial pseudoreplication in our dataset arising from multiple responses. Table S3. Best GLM models by exhaustive fit for the Meta Analysis model, Protected Areas (PA) model and Socio-Economic model. Table S4. Proportion of five highest ranked models for Meta Analysis model, PA model and the Socio-Economic model. Table S5. Best GLM model by exhaustive fit for two variables, the maximum distance to protected area boundary within studies, and the maximum distance between pair wise comparisons within studies. Figure S1. PRISMA flow diagram, depicting the flow of information through different phases of the search process conducted. Figure S2. Funnel plot of effect size standard error plotted against effect size for all inside-outside pairwise comparisons. Figure S3. Cumulative meta-analysis of the dataset sorted by precision, with effect sizes and 95% confidence intervals (n = 861). Appendix S1. PRISMA (Preferred Reporting Items for Systematic Reviews and Meta-Analyses) checklist. Appendix S2. Detailed descriptions of IUCN protected area management categories. (DOCX) [file pone.0105824.s001.docx]

**Supporting Materials:**

**Local scale comparisons of biodiversity as a test for global protected area ecological performance: a meta-analysis**

**Authors:** Bernard W. T. Coetzee^1,2,3^, Kevin J. Gaston^4^, Steven L. Chown^2^

**Affiliations:**

^1^Centre for Invasion Biology, Department of Botany and Zoology, Stellenbosch University, Stellenbosch, Western Cape, South Africa.

^2^School of Biological Sciences, Monash University, Melbourne, Victoria, Australia.

^4^Environment and Sustainability Institute, University of Exeter, Penryn, Cornwall, United Kingdom.

^3^Correspondence to B.W.T. Coetzee: email: bwtcoetzee@gmail.com.

**Table S1.** Orwin’s Fail safe N is 1238 to reach an overall effect size of 0.222, meaning that 1238 pair-wise comparisons with null results on average would have to be added to reduce the observed effect size by half [27,30] which even in that hypothetical event would nonetheless remain a positive value.

| **Overall effect size** | **Overall sample size** | **Target effect size** | **Orwin’s *Fail safe N*** |
| --- | --- | --- | --- |
| 0.444 | 861 | 0.222 | 1238 |

**Table S2.** Effect sizes determined by resampling one pairwise comparison per unit of study, per species, per country and per protected area (PA), to assess the potential spatial pseudoreplication in our dataset arising from multiple responses. These randomisations were repeated 10 000 times for each of these four parameters and the estimated mean and 95% confidence interval thereof compared to the overall effect size for all data for both pairwise comparisons inside and outside PAs and also for those within PAs only.

*The resampling by country for inside PA comparisons only used a Hunter-Schmidt estimator since the maximum likelihood estimator could not converge at such a low sample size (see [31]).

|  |  |  |  |  |  |
| --- | --- | --- | --- | --- | --- |
| **Comparison** | **Resample unit** | **Effect size** | **Lower CI** | **Upper CI** | **N** |
| *Inside Outside PA* | *All data* | *0.444* | *0.324* | *0.564* | *861* |
| Inside Outside PA | Study | 0.591 | 0.240 | 0.941 | 86 |
| Inside Outside PA | Species | 0.502 | -0.011 | 0.654 | 241 |
| Inside Outside PA | Country | 0.674 | -0.021 | 1.359 | 32 |
| Inside Outside PA | Protected Area | 0.827 | 0.317 | 1.337 | 57 |
| *Within PA only* | *All data* | *0.172* | *0.083* | *0.261* | *623* |
| Within PA only | Study | 0.194 | 0.004 | 0.385 | 43 |
| Within PA only | Species | -0.212 | -0.364 | -0.061 | 186 |
| Within PA only | Country* | 0.104 | -0.200 | 0.408 | 20 |
| Within PA only | Protected Area | 0.237 | 0.024 | 0.451 | 35 |
|  |  |  |  |  |  |

**Table S3.** Best GLM models by exhaustive fit for the Meta Analysis model, Protected Areas (PA) model and Socio-Economic model, respectively. The models were constructed across pairwise comparisons where data were available, and pairwise comparisons were excluded if explanatory data could not be obtained for them. Variables: *pa_iucn_cat* = protected area IUCN category; *area =* area of the PA in km^2^; *PA age* = establishment year of the PA; c*ontinen*t = continent in which the PA is embedded. *wgi* = World Governance Index; *gini* = Gini coefficient; *popsize* = country human population size; *gdp* = Gross Domestic Product. Interactions between terms are shown by “ : ” Visual inspection of residual plots [39], showed no trend which indicates that a linear model was approporiate (data not shown).

|  | **Meta Analysis model** | |  |
| --- | --- | --- | --- |
|  |  | |  |
|  | n = 861 | |  |
|  | Deviance explained | | 5.17% |
| Variables: | Slope | SE | P |
| **Intercept** | **3.68** | **0.002** | ******* |
| pa_iucn_cat | 0.008 | 0.002 | ******* |
| pa_iucn_cat:birds | 0.0007 | 0.0027 |  |
| pa_iucn_cat:herptiles | 0.0007 | 0.00379 |  |
| pa_iucn_cat:mammals | -0.0087 | 0.002667 | ****** |
| pa_iucn_cat:plants | -0.0108 | 0.00291 | ******* |
|  |  |  |  |
|  |  |  |  |
|  |  |  |  |
|  | **Protected Areas model** | |  |
|  |  | |  |
|  | n = 527 | |  |
|  | Deviance explained | | 25.03% |
| Variables: | Slope | SE | P |
| **Intercept** | **4.204** | **0.307** | ******* |
| continent.Australia | -0.136 | 1.561 |  |
| continent.Europe | 8.028 | 3.312 | ***** |
| continent.North America | 0.068 | 0.016 | ******* |
| continent.South America | 1.690 | 1.050 |  |
| latitude | -0.003 | 0.002 |  |
| area | 0.001 | 0.001 | ***** |
| PA age | -0.001 | 0.001 |  |
| area:longitude | -0.001 | 0.001 | ***** |
| continent.Asia:area | 0.001 | 0.001 | ****** |
| continent.Europe:area | -0.001 | 0.001 |  |
| continent.North America:area | -0.001 | 0.001 |  |
| continent.South America:area | -0.001 | 0.001 | ***** |
| continent.Asia:PA age | 0.001 | 0.001 |  |
| continent.Europe:PA age | -0.003 | 0.001 | ***** |
| continent.South America:PA age | -0.001 | 0.001 |  |
|  |  |  |  |
|  |  |  |  |
|  |  |  |  |
|  | **Socio Economic model** | |  |
|  |  | |  |
|  | n = 769 | |  |
|  | Deviance explained | | 7.41% |
| Variables: | Slope | SE | P |
| **Intercept** | **3.665** | **0.012** | ******* |
| gdp | 0.001 | 0.001 | ***** |
| gini | 0.001 | 0.001 | ***** |
| gdp:wgi | -0.001 | 0.001 | ****** |
| gini:popsize | -0.001 | 0.001 | ******* |
| wgi:popsize | 0.001 | 0.001 | ******* |
|  |  |  |  |

**Significance codes: *** = p < 0.0001; ** = p < 0.001 ; * = p < 0.05**

**Table S4.** Proportion of five highest ranked models for Meta Analysis model, PA model and the Socio-Economic model. One pairwise comparison per study was selected, and the respective GLM model fit as in table S3. We selected the highest ranked model based on the Akaike Information Criterion, and repeated this procedure 1000 times, to calculate the proportionally highest ranked model for each candidate dataset. Number and proportion of remaining models that were selected as the highest ranked model at least once is shown in italics. *wgi* = World Governance Index; *gini* = Gini coefficient; *popsize* = country human population size; *gdp* = Gross Domestic Product; *null* = Intercept only model, and see legend in table S3.

|  |  |  |
| --- | --- | --- |
| **Model type** | **Model formulae** | **Proportion as top ranked model** |
| Meta Analysis | Null | 0.529 |
| Meta Analysis | pa_iucn_cat | 0.362 |
| Meta Analysis | Metric | 0.071 |
| Meta Analysis | 1 + pa_iucn_cat + metric:pa_iucn_cat | 0.024 |
| Meta Analysis | 1 + metric + pa_iucn_cat + metric:pa_iucn_cat | 0.005 |
|  |  |  |
| *Meta Analysis* | *Remaining models (5)* | *0.009* |
|  |  |  |
|  |  |  |
| Protected Areas | area_km2 + pa_age + continent:area_km2 | 0.066 |
| Protected Areas | continent + pa_age + continent:pa_age | 0.064 |
| Protected Areas | continent + lat + pa_age + continent:pa_age | 0.058 |
| Protected Areas | continent + lat + area_km2 + pa_age + pa_age:area_km2 + continent:pa_age | 0.049 |
| Protected Areas | continent + pa_age | 0.047 |
|  |  |  |
| *Protected Areas* | *Remaining models (141)* | *0.716* |
|  |  |  |
|  |  |  |
| Socio-Economic | Wgi | 0.209 |
| Socio-Economic | Null | 0.151 |
| Socio-Economic | popsize:gini + wgi:popsize | 0.135 |
| Socio-Economic | gdp + wgi + popsize:gini + wgi:gdp + wgi:popsize | 0.069 |
| Socio-Economic | Gini | 0.054 |
|  |  |  |
| *Socio-Economic* | *Remaining models (61)* | *0.382* |
|  |  |  |

**Table S5.** Best GLM model by exhaustive fit for two variables, the maximum distance to protected area boundary within studies, and the maximum distance between pair wise comparisons within studies, meaning, within each study, the maximum distance between sampling points assigned to all points in that study. The models were constructed across pairwise comparisons where data were available. Only the distance between comparisons enters the model as an explanatory variable.

|  | | **Distance between pair-**  **wise comparisons** | | |  | |  |
| --- | --- | --- | --- | --- | --- | --- | --- |
|  | | n = 569 | | |  | |  |
|  | | Deviance explained | | | 1.00% | |  |
| Variables: | | Slope | | SE | P | |  |
| **Intercept** | | **3.67** | | **0.001** | ******* | |  |
| Distance between comparisons | | 0.001 | | 0.001 | ***** | |  |
|  | |  | |  |  | |  |
|  |  | |  | | |  | |

**Significance codes: *** = p < 0.0001; ** = p < 0.001 ; * = p < 0.05**


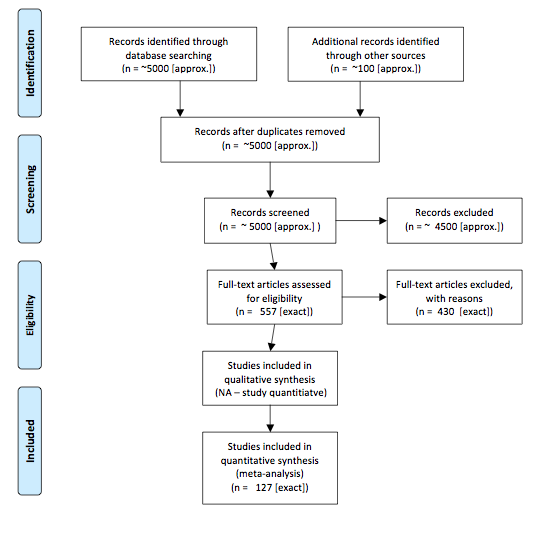


**Figure S1.** PRISMA flow diagram, depicting the flow of information through different phases of the search process conducted. A total of 127 papers met the sampling criteria both inside and outside PAs, of which 86 made comparisons inside and outside PAs.


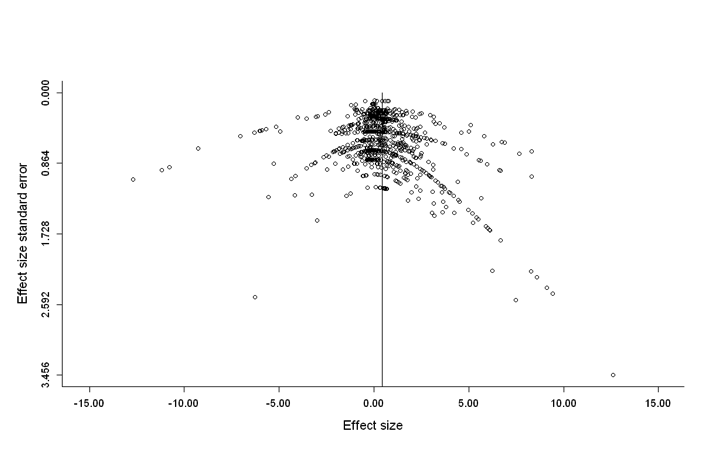


**Figure S2.** Funnel plot of effect size standard error plotted against effect size for all inside-outside pairwise comparisons. The relatively symmetrical plot suggests that those studies with small (or negative) effect sizes are not necessarily published at a lower frequency, so publication bias in our study can be considered slight [27,30,31]. The solid reference line indicates the overall effect size of 0.444 (n = 861). A funnel plot assumes that studies with the largest sample sizes will have lower standard error, and so will be near the average effect size, while studies with smaller sample sizes will be spread on both sides of the average effect size. Variation from this assumption can indicate bias. For example, positive asymmetry can indicate bias, in that those studies which found that PAs are effective where submitted and/or accepted for publication.

**
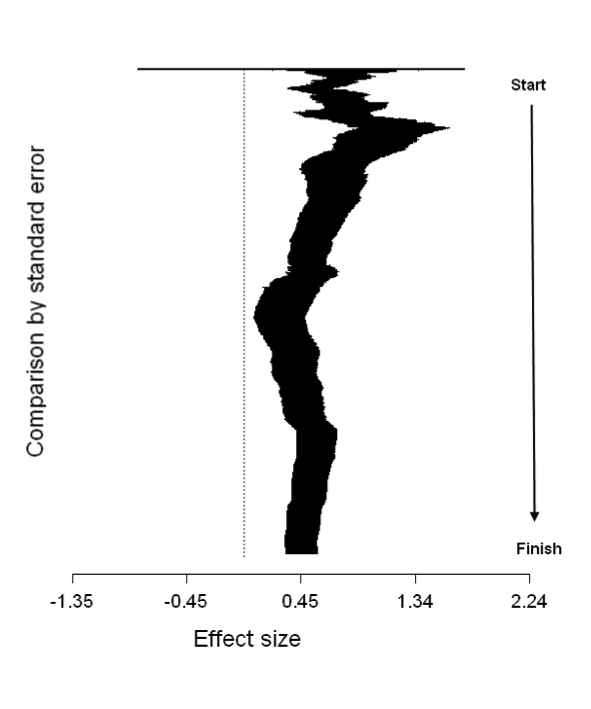
**

**Figure S3.** Cumulative meta-analysis of the dataset sorted by precision, with effect sizes and 95% confidence intervals (n = 861). The analysis starts with the comparison with the largest standard error, after which the comparison with the next largest standard error is added and the effect size is recalculated, and so continues iteratively until the analysis finishes with the comparison with the lowest standard error [27,30]. Essentially, the graph allows inspection of the development of the observed effect size with the addition of more precise data. The dotted line equals zero, or no effect. While the addition of the most imprecise studies does initially cause the cumulative effect size to decrease, it remains positive and does not overlap with zero at any point after the addition of the more precise studies, which reaffirms that the impact of bias in our study is negligible.

**Appendix S1.** PRISMA (Preferred Reporting Items for Systematic Reviews and Meta-Analyses) checklist. See: http://www.prisma-statement.org

| **Section/topic** | **#** | **Checklist item** | **Reported on page #** |
| --- | --- | --- | --- |
| **TITLE** | | |  |
| Title | 1 | Identify the report as a systematic review, meta-analysis, or both. | Title |
| **ABSTRACT** | | |  |
| Structured summary | 2 | Provide a structured summary including, as applicable: background; objectives; data sources; study eligibility criteria, participants, and interventions; study appraisal and synthesis methods; results; limitations; conclusions and implications of key findings; systematic review registration number. | Abstract |
| **INTRODUCTION** | | |  |
| Rationale | 3 | Describe the rationale for the review in the context of what is already known. | Introduction |
| Objectives | 4 | Provide an explicit statement of questions being addressed with reference to participants, interventions, comparisons, outcomes, and study design (PICOS). | Introduction |
| **METHODS** | | |  |
| Protocol and registration | 5 | Indicate if a review protocol exists, if and where it can be accessed (e.g., Web address), and, if available, provide registration information including registration number. | Methods |
| Eligibility criteria | 6 | Specify study characteristics (e.g., PICOS, length of follow-up) and report characteristics (e.g., years considered, language, publication status) used as criteria for eligibility, giving rationale. | Methods |
| Information sources | 7 | Describe all information sources (e.g., databases with dates of coverage, contact with study authors to identify additional studies) in the search and date last searched. | Methods |
| Search | 8 | Present full electronic search strategy for at least one database, including any limits used, such that it could be repeated. | Methods |
| Study selection | 9 | State the process for selecting studies (i.e., screening, eligibility, included in systematic review, and, if applicable, included in the meta-analysis). | Methods |
| Data collection process | 10 | Describe method of data extraction from reports (e.g., piloted forms, independently, in duplicate) and any processes for obtaining and confirming data from investigators. | Methods |
| Data items | 11 | List and define all variables for which data were sought (e.g., PICOS, funding sources) and any assumptions and simplifications made. | Methods |
| Risk of bias in individual studies | 12 | Describe methods used for assessing risk of bias of individual studies (including specification of whether this was done at the study or outcome level), and how this information is to be used in any data synthesis. | Methods |
| Summary measures | 13 | State the principal summary measures (e.g., risk ratio, difference in means). | Methods |
| Synthesis of results | 14 | Describe the methods of handling data and combining results of studies, if done, including measures of consistency (e.g., I^2^) for each meta-analysis. | Methods |

Page 1 of 2

| **Section/topic** | **#** | **Checklist item** | **Reported on page #** |
| --- | --- | --- | --- |
| Risk of bias across studies | 15 | Specify any assessment of risk of bias that may affect the cumulative evidence (e.g., publication bias, selective reporting within studies). | Methods, Supplementary File S1 |
| Additional analyses | 16 | Describe methods of additional analyses (e.g., sensitivity or subgroup analyses, meta-regression), if done, indicating which were pre-specified. | Methods, Supplementary File S1 |
| **RESULTS** | | |  |
| Study selection | 17 | Give numbers of studies screened, assessed for eligibility, and included in the review, with reasons for exclusions at each stage, ideally with a flow diagram. | Results |
| Study characteristics | 18 | For each study, present characteristics for which data were extracted (e.g., study size, PICOS, follow-up period) and provide the citations. | Supplementary Database S1 |
| Risk of bias within studies | 19 | Present data on risk of bias of each study and, if available, any outcome level assessment (see item 12). | Results; Supplementary File S1 |
| Results of individual studies | 20 | For all outcomes considered (benefits or harms), present, for each study: (a) simple summary data for each intervention group (b) effect estimates and confidence intervals, ideally with a forest plot. | Figures 2-3, Table 1 |
| Synthesis of results | 21 | Present results of each meta-analysis done, including confidence intervals and measures of consistency. | Fig 2, Fig 3 – All results in Table 1, Dataset S1 |
| Risk of bias across studies | 22 | Present results of any assessment of risk of bias across studies (see Item 15). | Figs S1,S3,Table 1 |
| Additional analysis | 23 | Give results of additional analyses, if done (e.g., sensitivity or subgroup analyses, meta-regression [see Item 16]). | Tables S2, S3 |
| **DISCUSSION** | | |  |
| Summary of evidence | 24 | Summarize the main findings including the strength of evidence for each main outcome; consider their relevance to key groups (e.g., healthcare providers, users, and policy makers). | Discussion |
| Limitations | 25 | Discuss limitations at study and outcome level (e.g., risk of bias), and at review-level (e.g., incomplete retrieval of identified research, reporting bias). | Discussion |
| Conclusions | 26 | Provide a general interpretation of the results in the context of other evidence, and implications for future research. | Discussion |
| **FUNDING** | | |  |
| Funding | 27 | Describe sources of funding for the systematic review and other support (e.g., supply of data); role of funders for the systematic review. | Acknowledgements |

*From:*  Moher D, Liberati A, Tetzlaff J, Altman DG, The PRISMA Group (2009). Preferred Reporting Items for Systematic Reviews and Meta-Analyses: The PRISMA Statement. PLoS Med 6(6): e1000097. doi:10.1371/journal.pmed1000097

For more information, visit: **www.prisma-statement.org**.

**Appendix S2.** Detailed descriptions of IUCN protected area management categories. Our analysis combined categories Ia and Ib into category 1.

Source (Online: accessed 24 Apr 2014):

**http://www.iucn.org/about/work/programmes/gpap_home/gpap_quality/gpap_pacategories/**

|  |  |  |
| --- | --- | --- |
| **Category** | **Name** | **Description** |
| Ia | Strict Nature Reserve | Category Ia are strictly protected areas set aside to protect biodiversity and also possibly geological/geomorphical features, where human visitation, use and impacts are strictly controlled and limited to ensure protection of the conservation values. |
| Ib | Wilderness Area | Category Ib protected areas are usually large unmodified or slightly modified areas, retaining their natural character and influence without permanent or significant human habitation, which are protected and managed so as to preserve their natural condition. |
| II | National Park | Category II protected areas are large natural or near natural areas set aside to protect large-scale ecological processes, along with the complement of species and ecosystems characteristic of the area, which also provide a foundation for environmentally and culturally compatible, spiritual, scientific, educational, recreational, and visitor opportunities. |
| III | Natural Monument or Feature | Category III protected areas are set aside to protect a specific natural monument, which can be a landform, sea mount, submarine cavern, geological feature such as a cave or even a living feature such as an ancient grove. They are generally quite small protected areas and often have high visitor value. |
| IV | Habitat/Species Management Area | Category IV protected areas aim to protect particular species or habitats and management reflects this priority. Many Category IV protected areas will need regular, active interventions to address the requirements of particular species or to maintain habitats, but this is not a requirement of the category. |
| V | Protected Landscape/ Seascape | A protected area where the interaction of people and nature over time has produced an area of distinct charcter with significant, ecological, biological, cultural and scenic value: and where safeguarding the integrity of this interaction is vital to protecting and sustaining the area and its associated nature conservation and other values |
| VI | Protected area with sustainable use of natural resources | Category VI protected areas conserve ecosystems and habitats together with associated cultural values and traditional natural resource management systems. They are generally large, with most of the area in a natural condition, where a proportion is under sustainable natural resource management and where low-level non-industrial use of natural resources compatible with nature conservation is seen as one of the main aims of the area |
|  |  |  |
